# Metabolic endotoxaemia related inflammation is associated with hypogonadism in overweight men

**DOI:** 10.1186/s12610-017-0049-8

**Published:** 2017-03-08

**Authors:** Kelton Tremellen, Natalie McPhee, Karma Pearce

**Affiliations:** 1 Department of Obstetrics Gynaecology and Reproductive Medicine, Flinders University, Bedford Park, South Australia; 2 School of Pharmacy and Medical Sciences, Division of Health Sciences, University of South Australia, Adelaide, 5001 South Australia; 3 Repromed, 180 Fullarton Road, Dulwich, South Australia

**Keywords:** Obesity, Hypogonadism, Endotoxin, Lipopolysaccharide (LPS), Testosterone, Leydig cell, Sertoli cell, Mots-Clés, Obésité, Hypogonadisme, Endotoxine, Lipopolysaccharide (LPS), Testostérone, cellule de Leydig, cellule de Sertoli

## Abstract

**Background:**

Obesity is associated with both impaired testosterone production and a chronic state of low grade inflammation. Previously it was believed that this inflammation was mediated by a decline in the immunosuppressive action of testosterone. However, more recently an alternative hypothesis (GELDING theory) has suggested that inflammation originating from the passage of intestinal bacteria into the circulation (metabolic endotoxaemia) may actually be the cause of impaired testicular function in obese men. The aim of this study is to investigate if metabolic endotoxaemia, as quantified by serum Lipopolysaccharide Binding Protein (LBP), is associated with impaired testicular endocrine function.

**Methods:**

A total of 50 men aged between 21 and 50 years (mean 35.1 ± 6.8 years) were assessed for adiposity (BMI, waist circumference and % body fat using bio-impedance), inflammatory status (serum CRP, IL-1β, IL-6, TNFα and LBP) and testicular endocrine function (serum testosterone, estradiol, AMH, LH and FSH). Statistical analysis was performed using Pearson correlation analysis, with log transformation of data where appropriate, and multi-variate regression.

**Results:**

Overall increasing adiposity (% body fat) was positively associated with metabolic endotoxaemia (LBP, r = 0.366, p = 0.009) and inflammation (CRP r = 0.531, p < 0.001; IL-6 r = 0.463, p = 0.001), while also being negatively correlated with serum testosterone (r = −0.403, p = 0.004). Serum testosterone levels were significantly negatively correlated with inflammation (CRP r = −0.471, p = 0.001; IL-6 r = −0.516, p < 0.001) and endotoxaemia (LBP) after adjusting for serum LH levels (p = −0.317, p = 0.03). Furthermore, serum IL-6 was negatively associated with AMH levels (r = −0.324, p = 0.023), with a negative trend between LBP and AMH also approaching significance (r = −0.267, p = 0.064).

**Conclusions:**

Obesity and its associated metabolic endotoxaemia helps initiate a pro-inflammatory state characterised by raised serum IL-6 levels, which in turn is correlated with impairment of both Leydig (testosterone) and Sertoli cell function (AMH). These results open up the potential for new treatments of obesity related male hypogonadism that focus on preventing the endotoxaemia associated chronic inflammatory state.

## Abstract (fr)

**Contexte:**

L’obésité est associée à la fois à une altération de la production de testostérone et à un état chronique d’inflammation de faible intensité. On pensait auparavant que cette inflammation était induite par une diminution de l’action immunosuppressive de la testostérone. Une hypothèse alternative plus récente (la théorie GELDING) a cependant suggéré que l’inflammation, qui a pour origine le passage de bactéries intestinales dans la circulation (endotoxémie métabolique), pourrait être en fait la cause de l’altération de la fonction endocrine du testicule chez les hommes obèses. Le but de la présente étude était de chercher si l’endotoxémie métabolique, évaluée par la quantification sérique de la Protéine Liant les Lypopolysaccharides (LBP), était associée à une altération de la fonction endocrine du testicule.

**Matériel et Méthodes:**

Un total de 50 hommes âgés de 21 à 50 ans (moyenne 35,1 ± 6,8 ans) ont été évalué pour l’adiposité (IMC, circonférence de la taille, et pourcentage de graisse corporelle par bio-impédance), le statut inflammatoire (CRP, IL-1beta, IL-6, TNFalpha et LBP sériques) et la fonction endocrine du testicule (testostérone, estradiol, AMH, LH et FSH sériques). L’analyse statistique des données a été réalisée par corrélation de Pearson - après transformation logarithmique des données quand nécessaire – et régression multivariée.

**Résultats:**

Une augmentation globale de l’adiposité (% de graisse corporelle) était positivement associée à l’endotoxémie métabolique (LBP, r = 0.366, p = 0.009) et à l’inflammation (CRP r = 0.531, p < 0.001; IL-6 r = 0.463, p = 0.001), tout en étant également corrélée négativement à la testostérone sérique (r = −0.403, p = 0.004). Les taux de testostérone sériques étaient, de façon significative, négativement corrélés à l’inflammation (CRP r = −0.471, p = 0.001; IL-6 r = −0.516, p < 0.001) et à l’endotoxémie métabolique (LBP) après ajustement sur les taux de LH sériques (p = −0.317, p = 0.03). L’IL-6 sérique était en outre négativement associée aux taux d’AMH (r = −0.324, p = 0.023), avec une tendance négative entre LBP et AMH approchant la signification (r = −0.267, p = 0.064).

**Conclusions:**

l’obésité et son endotoxémie métabolique associée favorisent l’initiation d’un état pro-inflammatoire caractérisé par une élévation des taux d’IL-6 sériques, état qui est en retour corrélé à une altération des fonctions des cellules de Leydig (testostérone) et de Sertoli (AMH). Ces résultats ouvrent la possibilité de nouveaux traitements de l’hypogonadisme masculin lié à l’obésité, centrés sur la prévention de l’endotoxémie associée à l’état inflammatoire chronique.

## Background

Obesity is an increasing public health concern with approximately one third of men from developed countries now being obese, and a further third overweight [1]. This increase in adiposity places men at increased risk of metabolic disorders such as diabetes, hypertension and hyperlipidaemia, but also impairs testosterone production and spermatogenesis [2–5]. Hypo-androgenism is associated with generalised depression and lethargy, plus the more specific sexual symptoms of erectile dysfunction and diminished desire [6], all significantly reducing men’s quality of life [2]. While weight loss usually results in normalisation of androgen levels and spermatogenesis [4, 7–9], unfortunately most obese men are unable to achieve this goal and therefore continue to suffer from the adverse effects of hypogonadism.

The current prevailing theory behind obesity related hypogonadism is centered on adipose tissue aromatase activity causing peripheral conversion of testosterone into estrogen [3, 4], which exerts a negative feedback effect on pituitary LH drive for testosterone production (central hypogonadism). In addition, “heating” of the testicles by the enveloping pelvic fat tissue [10], obesity related oxidative stress [11], and adipokines such as Leptin [12] may also play a role in perturbing testicular function in the obese man.

Multiple large epidemiological studies have reported significant positive correlations between obesity and various markers of inflammation (CRP, leukocyte count), yet negative associations between inflammation and serum testosterone [13–16]. This link between obesity, inflammation and declining testosterone has primarily been considered a result of withdrawal of testosterone’s immunosuppressive effect [13–15], although some previous investigators have also suggested that low grade inflammation may play a direct role in hypogonadism [16, 17].

Recently we proposed in the GELDING theory that inflammation triggered by the passage of gut bacterial endotoxin into the circulation of obese men may be responsible for impaired testosterone production [18]. The human intestine contains trillions of bacteria bearing the potent immune stimulant endotoxin (Lipopolysaccharide, LPS) [19, 20]. Under normal conditions nutrients selectively pass across the intestinal mucosal surface into the circulation, while preventing trans-migration of gut bacteria [21]. However, obesity and consumption of fatty food are both associated with a breakdown in intestinal mucosal integrity that allows passage of gut bacteria into the systemic circulation - so called “metabolic endotoxaemia” [19–22]. The resulting chronic state of low grade inflammation, typified by the production of pro-inflammatory cytokines such as Il-1β, TNFα and IL-6, has been reported to impair testicular steroidogenesis in both animal and human interventional studies [23–25].

Given the observed links between inflammation and hypogonadism, the underlying cause of inflammation in these obese men is of considerable scientific and clinical interest. Our recent proposal that endotoxin (lipopolysaccharide) derived from intestinal bacteria may trigger impaired gonadal function is novel, and if proven correct would open up new potential therapeutic strategies to combat obesity related hypogonadism [18]. Therefore the purpose of this study is to investigate the GELDING hypothesis that metabolic endotoxaemia related inflammation plays a significant role in impaired testicular function in overweight/obese men.

## Methods

### Study cohort

Participants were men aged between 18 and 50 years of age recruited from a private fertility clinic. Exclusion criteria were documented inflammatory or infective disease, the consumption of immune-suppressive medication (NSAID, corticosteroids, fish oil) or any hormonal therapy (aromatase inhibitors, clomiphene citrate, hCG or testosterone) in the last month.

### Assessment of adiposity

Height was measured to within 1 cm using a stadiometer, and weight (kg) and percentage body fat measured using bio-impedance digital scales (Tanita, UM051) to the nearest 0.1 kg and 0.1% respectively. Waist circumference was measured using a tape measure placed midway between the twelfth rib and the iliac crest to an accuracy of 0.5 cm. Body mass index (BMI) was calculated as body weight (kg) divided by height (m) squared. Men were classified as lean (BMI 18.5–24.9 kg/m^2^), overweight (BMI 25–29.9 kg/m^2^) or obese (BMI ≥ 30 kg/m^2^) as per WHO guidelines [26].

### Endocrine measurements

Blood was obtained by venepuncture between 8 and 10 am for all participants. Serum was analysed for estradiol, testosterone,, AMH, SHBG, FSH and LH using an automated chemiluminescence immunoassay (Cobas 6000 e 601, Roche Diagnostics, USA), with the detectable ranges for each hormone being 18.4–11010 pmol/L, 0.087–52.0 nmol/L, 0.071–164.2 pmol/L, 0.350–200 nmol/L,, 0.1–200 mIU/mL and 0.1–200 mIU/mL respectively. Calculated free testosterone was determined using the Vermeulen equation [27].

### Assessment of metabolic endotoxaemia and immune activation status

Metabolic endotoxaemia was quantified indirectly by Lipopolysaccaride Binding Protein (LBP) analysis using an ELISA according to the manufacturer’s guidelines (Hycult, Uden, Netherlands), with the minimum detectable concentration of LBP being 4.4 ng/mL. Direct measurement of endotoxin in plasma was not performed because of the well-documented inaccuracies inherent with these measurements [20, 28]. C-reactive protein (CRP) was measured in serum using an automated chemiluminesence machine (Integra 800, Roche Diagnostics, USA), with the limit of detection being 1 mg/L. Serum IL-1β, IL-6 and TNFα were analysed in duplicate serum samples using a multiplex immunoassay (ProcartaPlex kit, eBioscience). The detectable range for each of these cytokines was 0.21–860 pg/ml, 1.06 – 4340 pg/ml and 2.17 – 8900 pg/ml respectively.

### Statistical analysis

Statistical analyses were conducted using Graphpad Prism 7.01 (La Jolla, CA, USA) and IBM Statistical Product and Service Solution software, version 23 (SPSS Inc., Chicago, IL, USA). Data was expressed as mean (± standard deviation) when normally distributed, or as a median (inter-quartile range) when not normally distributed on formal testing. Correlations were assessed using the Pearson’s method, with log transformation of non-normally distributed data prior to statistical analysis. Multivariate analysis was performed with total and free testosterone as the primary outcomes of interest to determine possible confounders effecting testosterone levels. These confounders were then accounted for in a univariate analysis examining the statistical relationship between testosterone (total and free) and various markers of inflammation and body composition.

## Results

**Fig. 1:**
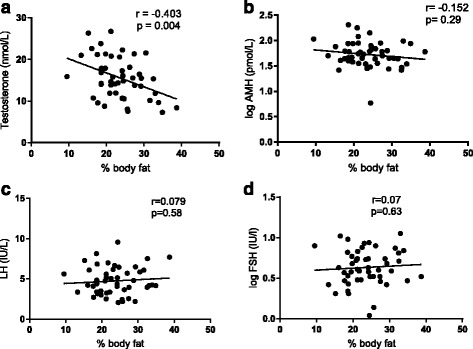
Relationship between adiposity (% body fat) and male reproductive hormones

The mean (± SD) age, BMI, percentage body fat and waist circumference of participants was 35.1 ± 6.8 years, 26.96 ± 3.5 kg/m^2^, 23.6 ± 6% and 93.2 ± 9.5 cm respectively. The endocrine and inflammatory status characteristics of participants are summarised in Table 1. The majority of participants were overweight (54%) or obese (16%), with only 30% being of lean BMI. Adiposity status, as assessed by BMI, waist circumference and percentage body fat, were all negatively correlated with both total and calculated free testosterone levels (Fig. 1, Table 2). Adiposity however was not significantly correlated with any other reproductive hormone (estradiol, LH, FSH, AMH) (Fig. 1, Table 2).

**Table 1:** Participant inflammatory and endocrine characteristics

Variable	Mean ± SD or Median (IQR)
CRP (mg/L)	1 (0.9–3)
IL-6 (pg/ml)	5.47 (4.29–6.5)
IL1β (pg/ml)	1.21 (1.06–1.31)
TNFα (pg/ml)	0.78 (0.63–0.92)
LBP (ng/ml)	10.39 (9.1–13.5)
Total Testosterone (nmol/L)	15.5 ± 5.0
Calculated Free testosterone (pmol/L)	298.3 ± 90.7
LH (IU/L)	4.8 ± 1.8
FSH (IU/L)	4.9 ± 2.4
AMH (pmol/L)	49.9 (37.7–77.3)
Estradiol (pmol/L)	77.0 ± 25.9

**Table 2:** Correlation matrix

	BMI (log)	Waist (cm)	Testo-sterone	E2	LH	FSH (log)	AMH (log)	SHBG	cFT	CRP (log)	LBP	Il-6 (log)	IL-1β (log)	TNFα (log)
Body fat (%)	.844	.898	−0.403	.156	0.08	0.07	-.152	-.402	-.338	.531	.366	.463	.123	.207
BMI (log)		.827	-.306	.124	0.04	.112	-.151	-.356	-.172	.498	.296	.379	.081	-.051
Waist (cm)			-.310	.173	-.013	0.05	-.178	-.357	-.247	.432	.266	.424	.076	.084
Testo-sterone				.334	.178	.012	-.128	.555	.700	-.471	-.244	-.516	-.253	-.191
E2					.052	.130	-.061	-.003	.218	-.007	.130	-.068	-.273	-.187
LH						.380	-.283	.178	.084	.127	.334	.179	-.04	-.027
FSH (log)							-.291	.208	-.172	.112	.140	.225	-.116	-.108
AMH (log)								-.129	-.018	-.112	-.267	-.324	.299	.012
SHBG									.137	-.253	-.207	-.344	-.082	-.035
cFT										-.238	-.217	-.385	-.143	-.207
CRP (log)											.369	.515	.302	0.015
LBP												.399	.111	-.151
Il-6 (log)													.266	.221
IL-1β (log)														0.61

Statistical analysis using Pearson correlation test. All values represent correlation coefficient value (r), with those reaching statistical significance (*p* < 0.05) being indicated in bold type

**Fig. 2:**
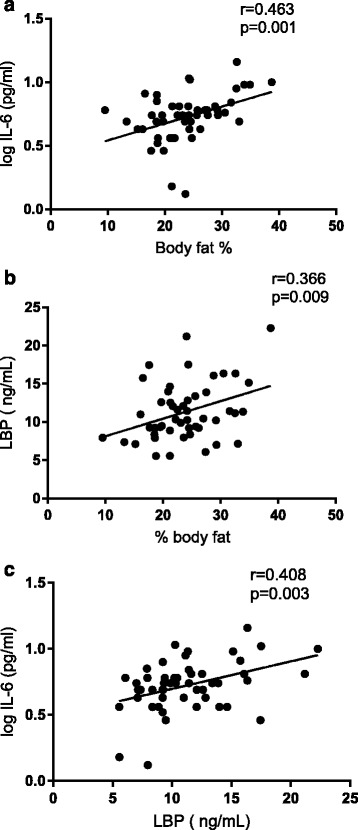
Relationship between adiposity (% body fat) and inflammation (LBP, IL-6)

**Fig. 3:**
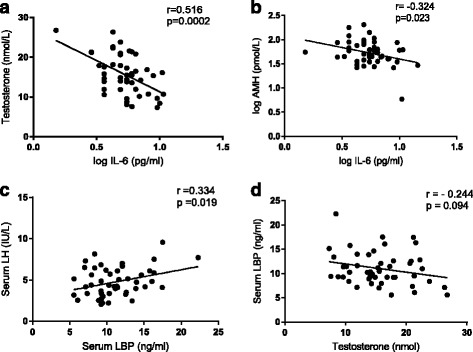
Relationship between inflammation (IL-6, LBP) and endocrine function (testosterone, LH and AMH)

In terms of inflammatory status, all three measures of adiposity (BMI, % body fat, waist circumference) were positively correlated with serum IL-6 (Fig. 1, Table 2) and CRP, but not with IL1β or TNFα (Table 2). Serum LBP was positively correlated with both adiposity (BMI and % body fat) and serum IL-6 (Fig. 2). In relation to CRP as an overall marker of inflammation, positive correlations were observed between both CRP and the cytokines IL1β and IL-6, but not with TNFα (Table 2). CRP was negatively correlated with total testosterone (*r* = −0.471, *p* = 0.001, Fig. 2), but not calculated free testosterone (*R* = −0.238, *p* = 0.11). Metabolic endotoxaemia (LBP) was positively correlated with serum LH (*r* = 0.334, *p* = 0.019) and significantly negatively correlated with testosterone concentration after adjusting for serum LH levels (*p* = −0.317, *p* = 0.03). A significant negative relationship between serum IL-6 and testosterone (*r* = −0.516, *p* < 0.001, Fig. 3) was also observed.

Multi-variant regression analysis to determine the predictors of total testosterone resulted in two significant models. In the strongest model IL-6, estrogen and SHBG explained 51% of the variation, F (3,41) = 7.5, *p* = 0.009, r^2^ = 0.51, while in the second model IL-6 and Estrogen explained 43% of the variation, F (2,42) =9.7, *p* = 0.001, r^2^ = 0.43. A further multi-variant analysis using free testosterone as the dependent variable resulted in 2 models. In model 1 the use of IL-6 alone explained 15% of the variation in free testosterone, F (1,44) = 9.0, *p* = 0.004, r^2^ = 0.15, whereas in model 2 IL-6 and estrogen explained 29% of the variation F (2,42) = 8.0, *p* = 0.001, r^2^ = 0.29.

In relation to Sertoli cell function, serum IL-6 was negatively associated with serum AMH (*r* = −0.324, *p* = 0.023, Fig. 3), with a negative trend between LBP and AMH approaching statistical significance (*r* = −0.267, *p* = 0.064). Serum LBP was not significantly correlated with the indirect marker of Sertoli cell function serum FSH.

## Discussion

The principal finding of this study is that male adiposity was associated with both metabolic endotoxaemia and an increase in serum IL-6, with this heightened inflammatory response being associated with a decline in both Leydig (testosterone) and Sertoli cell (AMH) function. Several previous studies have also reported a similar negative correlation between serum IL-6 and testosterone [16, 23, 29, 30], with the majority of these investigators suggesting that the observed increase in IL-6/inflammation with obesity was due to a withdrawal of testosterone’s immunosuppressive effect [13–15, 29, 30]. However, we believe that IL-6 may be directly inhibiting testicular function, thereby leading to a reduction in testosterone and AMH levels. This suggestion is consistent with earlier work in which the application of IL-6 to rodent Leydig cells in vitro resulted in a decline in testosterone production, due to the inhibition of key steroidogenic enzymes [31]. Furthermore, intra-venous administration of recombinant IL-6 to healthy men has been reported to result in a decline in serum testosterone [23]. Finally, it should be noted that administration of testosterone replacement therapy to hypogonadal men does not result in a decline in serum IL-6, thereby weakening the existing prevailing argument that a reduction in testosterone’s immunosuppressive action is responsible for increased IL-6 levels [32, 33]. As such, we believe it is more plausible that the negative relationship between testosterone and IL-6 is produced by the cytokine, directly inhibiting testosterone production.

Similar to our results, the NUMEVOX study of 229 overweight/obese men also reported a significant negative correlation between serum IL-6 and bioavailable testosterone [16]. This group initially hypothesised those inflammatory mediators such as IL-6 may produce central hypogonadism by up-regulating adipose aromatase activity, with the resulting estradiol reducing pituitary LH drive for testosterone production, since pro-inflammatory cytokines are known inducers of aromatase action. However, the NUMEVOX study actually found no significant relationship between estradiol and testosterone levels, and therefore it is unlikely that aromatisation is the primary mechanism of hypogonadism in these obese men. Our study also observed no correlation between BMI and serum estradiol (*r* = 0.124, *p* = 0.39) or LH (*r* = 0.04, *p* = 0.77), despite the presence of a significant negative correlation between BMI and serum testosterone (*r* = −0.306, *p* = 0.034), which also suggests that aromatisation is unlikely to be the dominant cause for obesity related hypogonadism. However, in our study cohort multi-variant analysis did identify estradiol as a significant variable effecting both total and free testosterone, thereby suggesting that estrogen does play some small part in obesity related hypogonadism.

Importantly, our observation of a positive correlation between endotoxaemia (LBP) and serum LH (*r* = 0.334, *p* = 0.019) suggests a peripheral (testicular) cause for obesity related hypogonadism, with a compensatory rise in LH release in response to falling testosterone production, rather than adipose aromatase/estradiol mediated central hypogonadism. A similar trend of an increase in serum LH accompanying a fall in serum testosterone following administration of rIL-6 to healthy male volunteers has previously been reported [23]. Interestingly, administration of the pro-inflammatory cytokine IL-2 has also been reported to reduce testosterone production in reproductive age men, primarily due to a combination of a reduction in basal LH levels and testicular responsiveness to LH [16]. However, since obesity is not associated with increased serum IL-2 levels [34], the use of IL-2 to replicate inflammatory processes seen in obesity is probably not an inappropriate model to study obesity related hypogonadism.

While our findings suggest that IL-6 has a greater inhibitory effect on the Leydig cells (decreased testosterone and increased LH) than Sertoli cell function, the observed decline in serum AMH also suggests some impairment of Sertoli cell function by IL-6. This finding is consistent with previous publications, with reductions in serum testosterone with increasing BMI being more prominent than associated declines in markers of Sertoli cell function, such as AMH or inhibin B [35]. These observations also make good physiological sense since the Leydig cells are present within the testicular interstitium, outside the blood-testis immunological barrier, and therefore are in intimate contact with interstitial macrophages and serum cytokines [31]. Therefore macrophages and their neighbouring Leydig cells will be exposed to any obesity related increase in serum IL-6, which may in turn impair testosterone production. However the Sertoli cells are contained behind the blood testis barrier [31], making them more resistant to the inhibitory action of circulatory IL-6. Secondly, as testosterone directly inhibits production of AMH [36], any obesity related reduction in testosterone production is likely to result in a reflex increase in AMH production, potentially masking any obesity related impairment in Sertoli cell function assessed by serum AMH.

This study is the first to provide supportive evidence for the GELDING theory by linking metabolic endotoxaemia with inflammation and impaired gonadal function in overweight and obese men. Our findings of a significant positive correlation between various markers of adiposity and LBP is consistent with prior reports and confirms that increasing adiposity is associated with metabolic endotoxaemia in our study cohort. Furthermore, the significant positive correlation between serum LBP and IL-6 suggests that metabolic endotoxaemia is likely to be a trigger for IL-6 release, with IL-6 then directly inhibiting testicular function. However we acknowledge that metabolic endotoxaemia is unlikely to be the sole cause of increased serum IL-6 in obese men. A recent study has confirmed that activated macrophages contained within adipose tissue also are a significant source of IL-6 production [37]. We believe that gut derived endotoxin exposure may provide the initial “spark” that both initiates and then maintains the “flames of inflammation” within adipose tissue macrophages- thereby maintaining high serum IL-6 levels. Our earlier report of an increase in macrophage activity in semen (seminal plasma neopterin) with increasing BMI is consistent with our belief that obesity related inflammation can extend to the male reproductive tract [11].

We acknowledge several potential weaknesses in our study. Firstly, this is only a pilot study of 50 men, with 16% of participants actually being obese. The recruitment of a larger sample size with a greater proportion of obese men is likely to produce more statistically rigorous results. Secondly, while we have reported very significant positive correlations between LBP and IL-6, and negative correlations between IL-6 and testosterone levels, the purely observational nature of our study does not enable us to draw definitive cause-effect relationships. However, given previous reports linking IL-6 exposure to impaired testosterone production [16, 23, 29, 30], we do believe that metabolic endotoxaemia related increase in IL-6 production is likely to play some role in impairing testicular function in obese men. We also acknowledge that LBP is only an indirect measure of endotoxaemia and the passage of gut bacteria derived endotoxin into the circulation. Therefore studies that directly quantify obesity related changes in intestinal permeability (e.g. sugar absorption testing) and levels of endotoxin in the circulation with markers of testicular function will be required to provide direct evidence for the GELDING theory [18]. Finally, our study did not assess symptoms of androgen deficiency, a worthwhile future endeavour, as male wellbeing, not serum testosterone, is the primary clinical end-point of interest.

A large body of scientific evidence has now linked low levels of testosterone in men with an increase in the incidence of cardiovascular disease [38, 39]. Previously this adverse association had been explained by the common co-morbidity of obesity, diabetes and hypertension in hypogonadal men- all known risk factors for cardiovascular disease. Interestingly, while testosterone replacement therapy (TRT) has been reported to improve both body composition (reduced fat and increased muscle mass), and glucose metabolism in hypogonadal men [40], the overwhelming majority of studies report that androgen replacement does not result in a reduction in cardiovascular disease, and even more alarmingly some studies have reported an increased cardiovascular event risk from TRT therapy [41]. Therefore could androgen deficiency be an epi-phenomenon- associated with cardiovascular disease but not the actual cause? Interestingly endotoxaemia related inflammation has been suggested to play a major role in the metabolic and cardiovascular complications of obesity [18, 20, 38, 39], and therefore it is possible that a more effective treatment for obese men is to target endotoxaemia related inflammation, rather than add back testosterone replacement. Resolution of endotoxaemia may improve men’s wellbeing by raising testosterone levels, while also reducing cardiovascular events. Of course at present this is still a hypothetical concept, but one well worth exploring in the future.

According to the GELDING hypothesis [18], the key to effective treatment of obesity related hypogonadism is to improve the barrier function of the intestine, thereby preventing trans-migration of endotoxin from the bowel lumen into the circulation and lowering inflammation known to impair testicular function. One potential treatment that targets this process is the ingestion of probiotic “good bacteria”. These beneficial microbes improve gut wall integrity by releasing Short Chain Fatty Acids (SCFA) that nourishes the adjacent mucosa, improving its barrier integrity. Secondly probiotic bacteria complete for nutrients with potentially pathogenic gram negative bacteria, thereby reducing the endotoxin load within the intestine [42]. A recent study supporting this “gut health” approach reported that ingestion of probiotic *lactobacillus reuteri* bacteria resulted in an increase in Leydig cell density and improvements in both serum testosterone and spermatogenesis in mice compared to controls [43]. This beneficial effect of probiotics on male reproductive function is most likely mediated by the immune system, since blocking inflammation also resulted in a similar improvement in testosterone production and spermatogenesis. To date no such studies have been conducted in men, but the results of this study certainly support the possibility that this therapeutic approach may be fruitful to investigate in the future.

## Conclusions

The results of this study confirm that adiposity is associated with increasing levels of inflammation (serum CRP, IL-6) and metabolic endotoxaemia (LBP), while also being associated with a significant reduction in serum testosterone, independent of changes in serum gonadotrophins (LH, FSH). Interestingly, serum IL-6 levels were also negatively correlated with both serum testosterone and AMH, raising the possibility that this key pro-inflammatory cytokine may play a direct role in impairing Leydig and Sertoli cell function. Furthermore, the observed positive correlation between adiposity, metabolic endotoxaemia (LBP) and serum IL-6 supports the possibility that endotoxin exposure in obese men may be a significant trigger for increased serum IL-6 production, which then in turn impairs testicular function. While this observational study cannot prove a direct causal link between obesity, increased endotoxin exposure and male hypogonadism (GELDING theory), it is the first human study to provide indirect evidence supporting this link. Furthermore, these results open up the potential for future new treatments for obesity related male hypogonadism that focus on preventing metabolic endotoxaemia and its associated chronic inflammatory state.

## Abbreviations

AMH: Antimullerian Hormone
BMI: Body Mass Index
CRP: C-reactive protein
FSH: Follicle Stimulating Hormone
GELDING: Gut Endotoxin Leading Decline IN Gonadal function
IL-1β: Interleukin 1β
IL-6: Interleukin 6
LBP: Lipopolysaccharide Binding Protein
LH: Luteinizing Hormone
LPS: Lipopolysaccharide
NSAID: Non-steroidal anti-inflammatory drugs
SCFA: Short chain fatty acids
TNFα: Tumour Necrosis Factor alpha
TRT: Testosterone replacement therapy
WHO: World health organisation
